# Supplementary material for: A simple method of equine limb force vector analysis and its potential applications
Source: PeerJ. 2018 Feb 21;6:e4399. doi: 10.7717/peerj.4399 (PMC5827015; doi:10.7717/peerj.4399)
Supplement: Supplemental Information 1 [file peerj-06-4399-s001.docx]

**Supplementary Information**

A sensitivity analysis was performed upon the GRF data to interrogate the sensitivity of VecMag and VecAng variables to the threshold used for identifying the stance phase. Firstly, for the group data additional events were created 1 frame before and 1 frame after footstrike and lift off for all trials. The magnitude of GRF was recorded at these new events to determine whether the event would have occurred at the same time or a different time if a different threshold was used to identify the event (50, 100 and 150N in accordance with Starke and Clayton, 2015) .

For all four limbs footstrike would have occurred at the same time for all thresholds, so footstrike was not influenced by the threshold as the vertical force rose rapidly on contact. Lift off was influenced by the threshold and this was more evident in the hindlimb compared to the forelimb (see Table S1, Sensitivity analysis.xls), which is in agreement with Starke and Clayton (2015).

**Table S1: The sensitivity of using a different threshold to determine lift off for the group of horses. The reference threshold is 50 N (used in the study for identification of both events). The effect of using 100N and 150N thresholds are then quantified by the number of trials using an earlier lift off event and the mean ± s.d. time difference between 50N and 100N and 50N and 150N events.**

| **Limb** | **Threshold (N)** | **# trials using earlier event** | | **Time difference (ms)** |
| --- | --- | --- | --- | --- |
|  |  | **1 frame** | **2 frames** |  |
| Left fore | 100 | 7 |  | 1.9 (3.6) |
|  | 150 | 14 |  | 3.9 (4.2) |
| Right fore | 100 | 10 |  | 2.7 (4.0) |
|  | 150 | 17 |  | 4.7 (4.2) |
| Left hind | 100 | 18 |  | 5.0 (4.1) |
|  | 150 | 18 | 8 | 9.4 (5.2) |
| Right hind | 100 | 12 |  | 3.3 (4.2) |
|  | 150 | 21 | 4 | 8.1 (4.6) |

Based on these findings, events were created 1 and 2 frames before right hindlimb lift off for each of the 8 trials of the individual horse. VecMag and VecAng were re-calculated using the two new events (Table S2). Vertical and longitudinal plots of the GRF profiles for each of the thresholds were created in Visual 3D for illustrative purposes (see Figure S1). Paired samples t-tests were performed in SPSS to establish whether the difference in threshold was significant within trials (p<.05).

**Table S2: Calculated mean (s.d.) for VecMag (N/kg) and Vec Ang (deg) for the right hindlimb based on events created 1 and 2 frames earlier than lift off and their corresponding vertical force magnitudes (N). Significant differences (p<.05) are denoted with an asterisk*.**

|  | **Vec Mag (N/kg)** | **Vec Ang (deg)** | **Vertical GRF (N)** |
| --- | --- | --- | --- |
| 50 N threshold (reference) | 5.15 (0.27)* | 1.51 (0.74) |  |
| 1 frame earlier | 5.35 (0.32)* | 1.51 (0.75) | 92.8 (23.3) |
| 2 frames earlier | 5.56 (0.29)* | 1.51 (0.75) | 168.5 (52.9) |

Significant differences were found for VecMag between all paired combinations, all significant at p<.01. No significant differences were found for VecAng paired combinations.

The results indicate that care must be taken in defining the threshold for stance phase identification, particularly in the hindlimbs when considering VecMag and other time-based variables. For this manuscript a 50N threshold was used, which has been used in previously, although other authors have used greater thresholds (see Starke and Clayton (2015) for a fuller examination of event detection methods. As VecMag was designed to encapsulate the entire force profile we opted to use a lower threshold that included the extent of the forces produced. Further work with a greater population of horses and different surfaces may examine the efficacy of this decision. In contrast, VecAng is largely independent of the event threshold, which highlights the potential use of this variable as an overall description of the longitudinal GRF effect.

**References**

Starke, S and Clayton HM. 2015. A universal approach to determine footfall timings from kinematics of a single foot marker in hoofed animals. *PeerJ* 3:e783; DOI 10.7717/peerj.783

**Figure Legends**

Figure S1: The effect of using a different threshold on ground reaction force traces normalized to 101 points. A) Mean vertical and B) mean longitudinal ground reaction forces for horse 10 (N/kg). Black: 50 N threshold; blue: 100 N threshold; red: 150 N threshold.
