# Supplementary figures and images for: A simple method of equine limb force vector analysis and its potential applications
Source: PeerJ. 2018 Feb 21;6:e4399. doi: 10.7717/peerj.4399 (PMC5827015; doi:10.7717/peerj.4399)

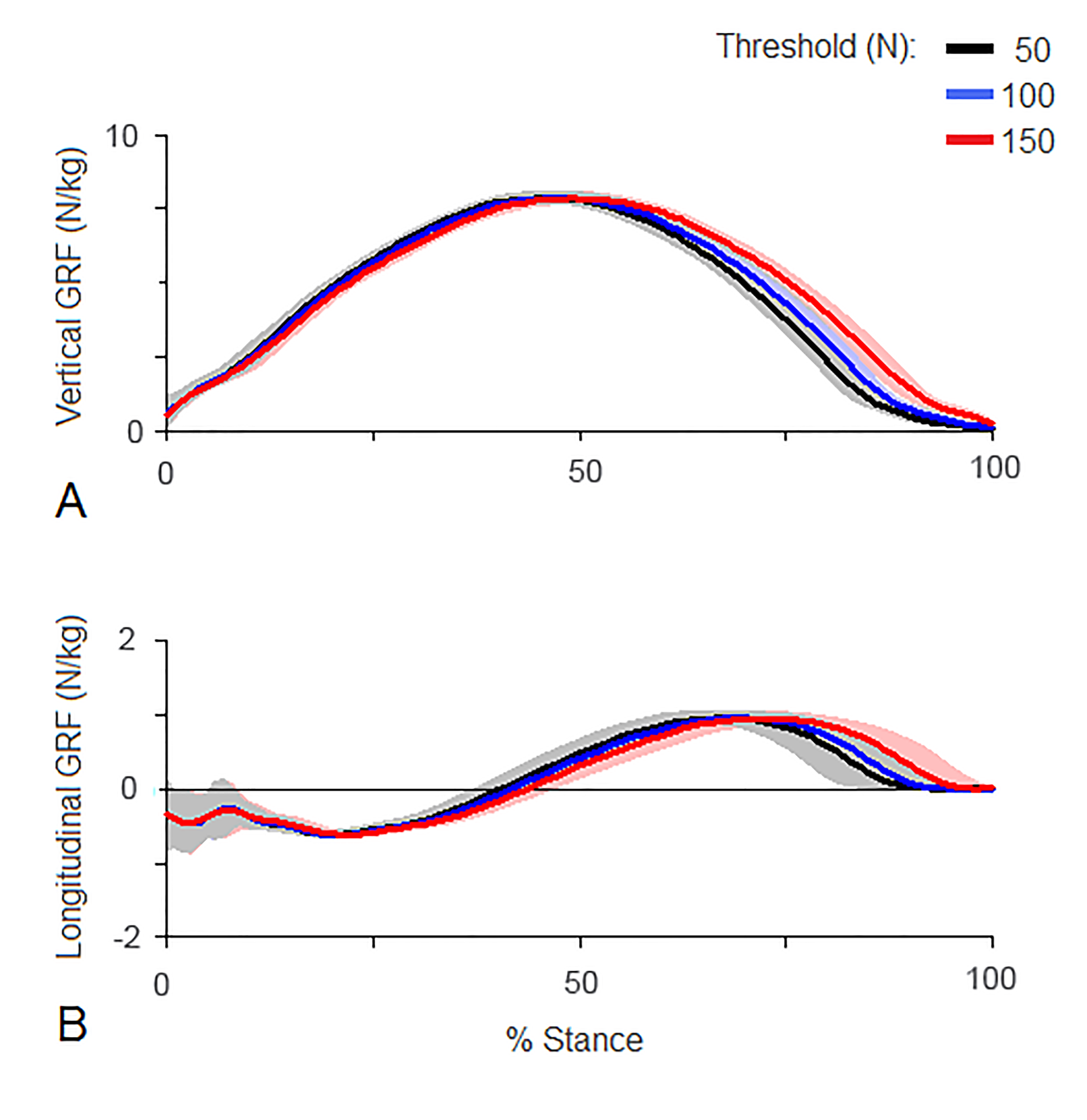

Supplement: Figure S1 — (A) Mean vertical and (B) mean longitudinal ground reaction forces for horse 10 (N/kg). Black: 50 N threshold; blue: 100 N threshold; red: 150 N threshold. [file peerj-06-4399-s002.png]
